# Efficacy and safety of mTOR inhibitors (rapamycin and its analogues) for tuberous sclerosis complex: a meta-analysis

**DOI:** 10.1186/s13023-019-1012-x

**Published:** 2019-02-13

**Authors:** Min Li, Ying Zhou, Chaoyang Chen, Ting Yang, Shuang Zhou, Shuqing Chen, Ye Wu, Yimin Cui

**Affiliations:** 1 Department of Pharmacy, Peking University First Hospital, 6 Dahongluochang Street, Xicheng District, Beijing, 100034 China; 2 Department of Pharmacy Administration and Clinical Pharmacy, School of Pharmaceutical Science, Peking University, Beijing, China; 3 Department of Neurology, Peking University First Hospital, Beijing, China

**Keywords:** mTOR inhibitors, Tuberous sclerosis complex, Meta-analysis

## Abstract

**Background:**

The treatment of tuberous sclerosis complex (TSC) using mammalian target of rapamycin (mTOR) inhibitors is clinically promising. The aim of the present study was to evaluate the efficacy and safety of mTOR inhibitors for improving the clinical symptoms of TSC.

**Methods:**

We performed a systematic search of major electronic databases (PubMed, EMBASE, Cochrane Library and WanFang, CNKI, and VIP databases) to identify randomized controlled trials (RCTs) and quasi-randomized studies from the date of database inception to November 2017; the Chinese Food and Drug Administration and *clinicaltrials.gov* were also searched for unpublished studies. The endpoints of the study were the tumor response rate and seizure frequency response rate (the proportion of patients achieving a ≥ 50% reduction relative to the baseline). Two researchers screened articles, assessed the risk of bias and extracted data independently. The included RCTs were analyzed using RevMan 5.3, which was provided by the Cochrane Collaboration.

**Results:**

Compared with the placebo, mTOR inhibitors significantly reduced tumor volume in both angiomyolipoma (AML) (RR = 24.69, 95% CI = 3.51,173.41, *P* = 0.001) and subependymal giant cell astrocytoma (SEGA) (RR = 27.85, 95% CI = 1.74,444.82, *P* = 0.02). Compared with the placebo, mTOR inhibitors significantly reduced seizure frequency (RR = 2.12, 95% CI = 1.41,3.19, *P* = 0.0003). Regarding safety, compared with patients who did not receive mTOR inhibitors, those who did had a higher risk of suffering stomatitis (RR = 3.20, 95% CI = 1.49,6.86, *P* = 0.003). In contrast, patients who did and did not receive mTOR inhibitors experienced similar adverse events, such as upper respiratory tract infections (RR = 1.08, 95% CI = 0.81,1.45, *P* = 0.59) and nasopharyngitis (RR = 0.86, 95% CI = 0.60,1.21, *P* = 0.38).

**Conclusion:**

In view of the efficacy and safety associated with tumor and seizure frequency in the TSC patients, mTOR inhibitors is a good therapeutic choice. Unlike the risks of upper respiratory tract infections and nasopharyngitis, mTOR inhibitors seem to increase the risk of stomatitis, mostly grade 1 and 2.

## Introduction

Tuberous sclerosis complex (TSC) is an orphan disease that affects many organ systems to varying degrees and is typically characterized by benign tumors of the skin (facial angiofibroma), brain (subependymal giant cell astrocytoma), kidneys (angiomyolipoma), heart (rhabdomyoma), lungs (lymphangioleiomyomatosis) and retina (optic nerve tumor). In addition, TSC can also cause cognitive nerve deficits and behavioral and developmental disorders, such as epilepsy. The estimated prevalence of TSC in recent studies falls in the range of 1/6000 to 1/10,000 [1]. The underlying molecular etiology of TSC is explained as the abnormal activation of mTORC1 (mTOR complexes 1) caused by the genetic mutation of TSC1 [2] or TSC2 [3], which leads to uncontrolled cellular proliferation via the promotion of protein synthesis and then stimulates benign tumor growth in many systems [4]. Genetic testing for TSC1 and TSC2 was included in the diagnostic criteria developed at the 2012 International TSC Consensus Conference [5]. Based on this pathogenic mechanism, rapamycin and its derivatives have been considerd as a new therapy for TSC, and they have recently received extensive attention at home and abroad.

As TSC has several highly diverse clinical symptoms, there are a number of different therapies for it. For acutely symptomatic subependymal giant cell astrocytoma (SEGA) in the brain, surgical resection is the recommended first-line therapy, while medical therapy with mTOR inhibitors is recommended for growing but asymptomatic SEGA. For asymptomatic and growing angiomyolipoma (AML) larger than 3 cm in diameter, therapy with mTOR inhibitors may be the most effective therapy according to some short-duration studies. There are special therapies for medically refractory epilepsy in TSC, such as epilepsy surgery and vagus nerve stimulation. For patients without clinical manifestations, there is no recommended therapy except traditional medicine. Furthermore, there is insufficient evidence to recommend therapy for TSC-associated skin lesions [6].

Rapamycin (sirolimus) is a macrolide compound isolated in 1975 from *Streptomyces hygroscopicus* in a soil sample from Easter Island. Everolimus (RAD001) is derived from rapamycin and has substantially more favorable pharmacokinetic characteristics [7], with better absorption, greater oral bioavailability [8], faster steady state levels after initiation and quicker elimination after discontinuation [9, 10]. Rapamycin and everolimus bind to FKBP12 (FK 506-binding protein of 12 kDa) to prevent mTOR from activating mTORC1 abnormally [11] and then control cellular proliferation to stop benign tumor growth.

Due to this mechanism, several individual case reports, small case series and open-label clinical trials [12–14] indicated that mTOR inhibitors could reduce tumor growth. In addition, there were some preclinical studies [15] and prospective studies [16] that suggested mTOR inhibitors could be a novel epilepsy treatment in patients with TSC. Currently, medical therapy has replaced surgery as the recommended therapeutic method for patients with SEGAs and AMLs. Although everolimus was approved by the FDA for renal AML and SEGA related to TSC in 2009 [17], the efficacy and safety of mTOR inhibitor therapy for other clinical symptoms in patients with TSC remain unclear. There are some views that no difference was observed between mTOR inhibitors and other therapies in TSC therapy. A single-center retrospective study [18] found that rapamycin had no effect on seizure frequency. According to a case report by Sparagana SP, rapamycin therapy resulted in an improvement in the patients’ SEGA but exerted no effect on optic nerve tumor [19]. Furthermore, in the meta-analysis performed by Sasongko TH et al [20], the literature search only extended until March 2016; however, two additional RCTs [21, 22] have since been published, providing more data about the use of mTOR inhibitors in patients with TSC. Therefore, we integrated all relevant randomized control trials to update the conclusions about the efficacy and safety of mTOR inhibitors for the treatment of TSC.

## Method

### Search strategy

Relevant studies were searched in the following six databases: PubMed, EMBASE, Cochrane Library, WanFang, CNKI and VIP. The search was limited to English and Chinese language publications that were published prior to November 10, 2017 (Subsequent searches were performed up to December 09, 2018). The search was performed with Medical Subject Headings (MeSH) and free text terms. The major search terms were ‘tuberous sclerosis complex’, ‘TSC’, ‘mTOR inhibitor’, ‘rapacymin’ and ‘everolimus’ in English and ‘jie jie xing ying hua zheng’, ‘mTOR yi zhi ji’, ‘lei pa mei su’ and ‘yi wei mo si’ in Chinese. We also scanned the references of the articles that met the eligibility criteria. The Chinese Food and Drug Administration database and *clinicaltrials.gov* were searched for unpublished studies.

### Study selection

The first filtering was performed to exclude articles that were clearly irrelevant. The abstracts of the remaining articles were screened to identify potentially relevant studies. The full texts of each article identified as potentially relevant during the abstract screening were reviewed and evaluated by two authors independently to select the studies for inclusion in the meta-analysis. When opinions differed, a discussion was conducted with a third reviewer. With regard to several articles that all related to the same study, we included the latest publication with the most complete data in the meta-analysis.

RCTs that assessed the efficacy and safety of mTOR inhibitors in patients with TSC were included in the present meta-analysis. The inclusion criteria were as follows: (a) subjects were TSC patients; (b) the RCTs compared the efficacy and safety of mTOR inhibitors with a placebo or no treatment; (c) the trials reported at least one outcome measure, including tumor response rate (the proportion of patients achieving a ≥ 50% reduction in tumor volume relative to the baseline) and seizure frequency response rate (the proportion of patients achieving a ≥ 50% reduction in seizure frequency relative to the baseline); and (d) oral administration was used in the trials.

### Assessment of risk of bias and data extraction

We used *the Cochrane Handbook* [23] to assess the risk of bias in each study. Each study was examined on the basis of sequence generation, allocation concealment, incomplete outcome data, selective outcome reporting, the blinding of patients and personnel and the blinding of the outcome assessment. We categorized these studies as have a ‘low risk’, ‘high risk’ or ‘unclear risk’ of bias.

Independent data selection, extraction and evaluation were performed by two authors separately. The extracted data were the therapy period, follow-up period, sample size, the subjects’ baseline characteristics, the subjects’ demographic characteristics, the subjects’ disease characteristics, the main efficacy findings, and the prevalence of adverse events (AEs). When any disagreements occurred regarding the data extraction, the two authors performing the data extraction reached a consensus after discussion or mediation by a third reviewer.

### Statistical analysis

We performed the statistical analyses with Review Manager (RevMan), Version 5.3. We stratified the studies according to outcome measure. Between-study heterogeneity was tested using the I^2^ index. If I^2^ was > 50%, there was substantial heterogeneity, and a random effects model was adopted for the meta-analysis to resolve the heterogeneity. When I^2^ was < 50%, the fixed effects model was used. Dichotomous data were calculated as risk ratios (RRs) and 95% confidence intervals (CIs). If a *P*-value was < 0.05 and the 95% CIs of the RRs did not cross 1.00, the results were regarded as statistically significant.

## Outcome

### Results

#### Studies included in the meta-analysis

**Fig. 1:**
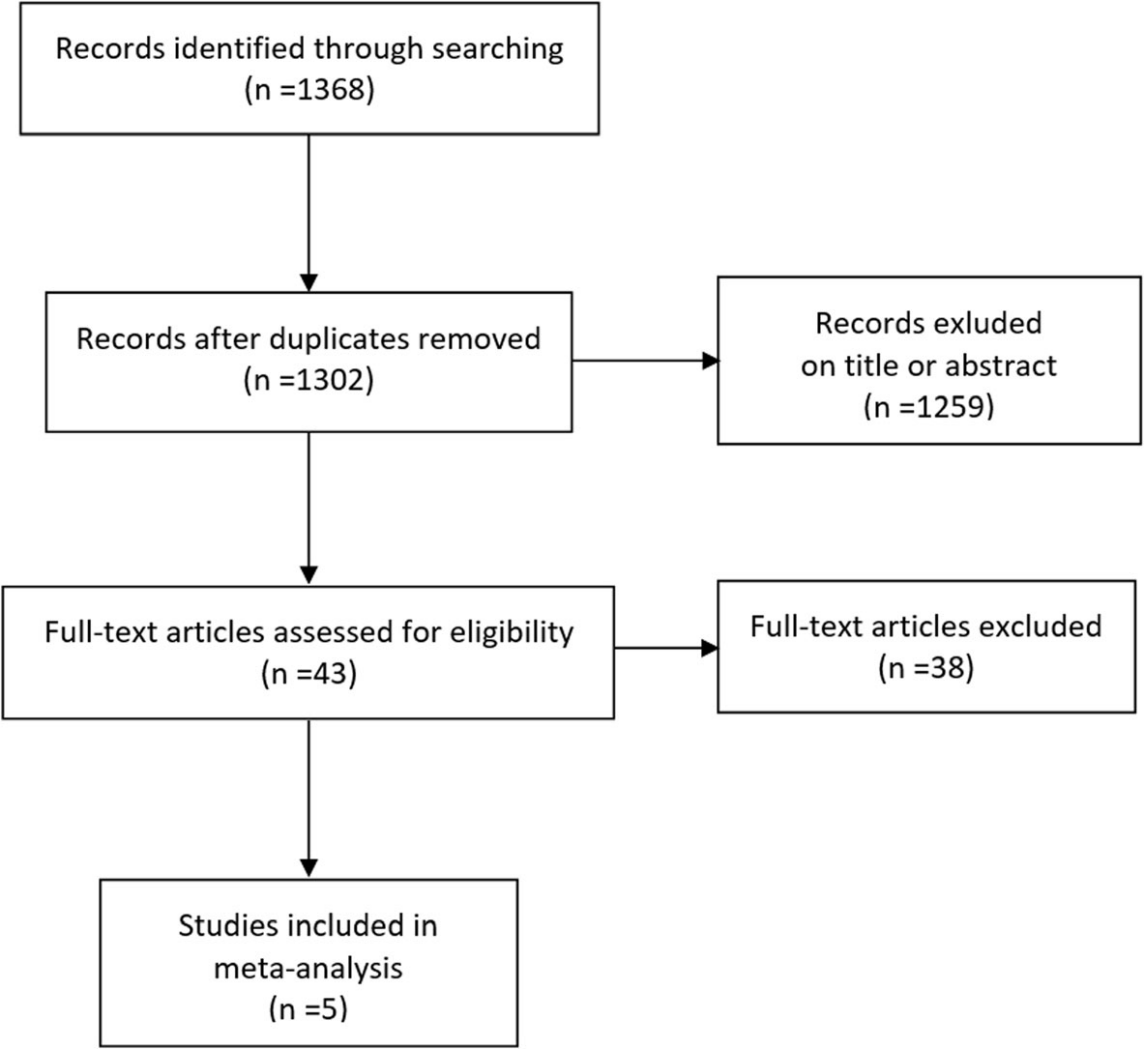
Flow diagram of study selection

In total, 1368 articles were identified, of which 66 articles were duplicates (*n* = 1302). Following the review of titles and abstracts, 1259 articles were excluded. The remaining 43 articles were reviewed in detail. Of these, 19 studies were study designs that did not meet the inclusion criteria, 13 included articles from the same trials, 4 did not match the purpose of the meta-analysis and 2 did not report the desired outcomes. Four RCTs [24–26] described as double-blinded, randomized, placebo-controlled studies and one [22] described as an open-label, add-on study were eligible for inclusion in this meta-analysis, with variable lengths of study duration. The process of article selection is illustrated in Fig. 1. The main study characteristics of the included studies are presented in Table 1. The number of patients in these trials ranged from 23 to 366, and the total number of patients was 671.

**Table 1:** Characteristics of the included studies in the meta-analysis

Study	Patients		Intervention	Comparison	Therapy duration	Inclusion criteria	Outcomes measures	Follow-up	Sample locations
	Number	Age (years)							
Franz 2013 [24] NCT00789828	78:39	0–65	Everolimus (orally, starting dose 4.5 mg/m^2^ per day)^a^	Placebo	6 months	TSC with one target SEGA (≥1 cm^3^)	The tumor response	4–5 years	Multicenter
Bissler 2013 [25] NCT00790400	79:39	18–61	Everolimus (orally 10 mg per day)	Placebo	6 months	TSC with at least one AML (≥3 cm^3^)	AML tumor volume response	4–5 years	Multicenter
French 2016 [21] NCT01713946	119:117:130	2–65	Everolimus (orally 3–9 mg/m^2^ per day) ^b^	Placebo	26 weeks	TSC and therapy-resistant seizures	Response rate of seizure frequency	1 years	Multicenter
Krurger 2017 [26] NCT01289912	32:15	6–21	Everolimus (orally 4.5 mg/m^2^ per day)	Placebo	6 months	TSC with a baseline verbal, performance or overall IQ score ≥ 60	TAND features	1 months	Two-center
Overwater 2016 [22] NTR3178	23:23	1.8–10.9	Sirolimus (orally, starting dose 1 mg/mL)^c^	Crossover design	6 months	TSC with at least 1 epileptic seizure per week and resistant to at least 2 AEDs	Response rate in seizure frequency	6 months	The Netherlands

*TSC* tuberous sclerosis complex, *SEGA* subependymal giant cell astrocytoma; response in tumor volume, (a ≥ 50% reduction relative to the baseline in SEGA or AML); response rate (the proportion of patients achieving a ≥ 50% reduction relative to the baseline in seizure frequency)

^a^ Orally, at a starting dose of 4.5 mg/m^2^ per day, subsequently adjusted to attain blood trough concentrations of 5–15 ng/mL

^b^ For patients younger than 10 years, the starting dose of everolimus was 6 mg/m^2^ per day for those not receiving CYP3A4/PgP inducers and 9 mg/m^2^ per day for those receiving CYP3A4/PgP inducers; for patients aged 10–18 years, the equivalent doses were 5 mg/m^2^ per day and 8 mg/m^2^ per day, respectively, and for those older than 18 years, they were 3 mg/m^2^ per day and 5 mg/m^2^ per day, respectively [21]

^c^ Starting from 1 mg/mL sirolimus orally, the dose was adjusted based on body weight and blood trough levels

#### Risk of bias

**Fig. 2:**
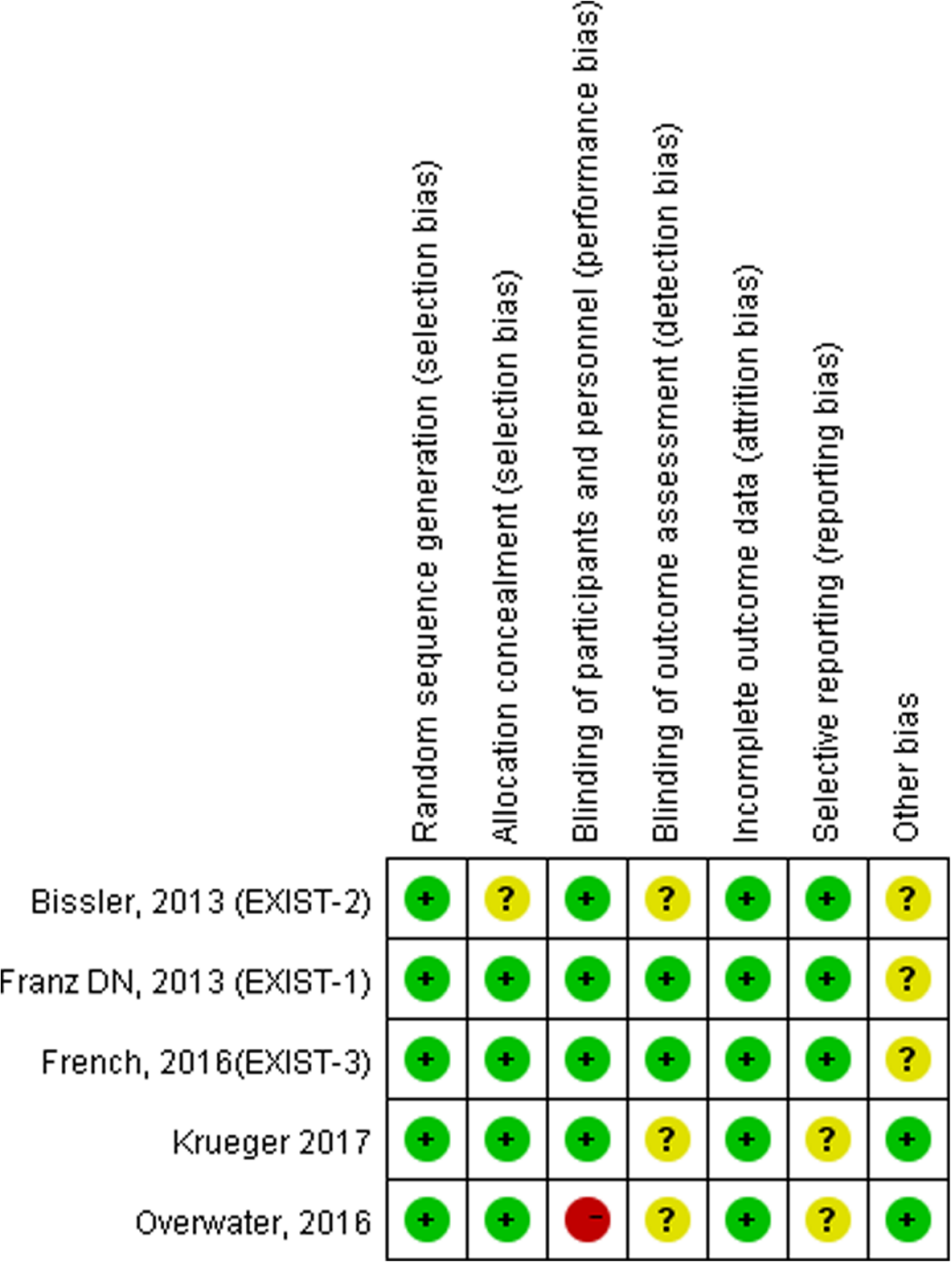
Risk of bias summary

Specific information regarding the bias in the included RCTs is summarized in Fig. 2. Studies performed by Bissler were devoid of details regarding the allocation concealment. Study personnel other than neuropsychologists and neurophysiologists were not blinded in the Overwater trial, so it was judged to have a high risk of performance bias. In addition, blinding for the outcome assessment and selective reporting were not reported in the Overwater trial. In the Franz, Bissler and French studies, it was noted that authors who are employees, stock owners or consultants of the funder (Novartis) were involved in the study design, discussion, research, overseeing of data collection and data analysis and interpretation; we assessed this as an unclear risk of bias. The Krurger study did not provide the reasons for the discontinuation of treatment by some patients, which meant the study had an unclear risk of reporting bias.

### Efficacy evaluation

We set the response rates as the outcome measures, including the tumor response rate and seizure frequency response rate. The tumor response rate was defined as the proportion of patients achieving a ≥ 50% reduction in tumor volume relative to the baseline. The seizure frequency response rate was defined as the proportion of patients achieving a ≥ 50% reduction in seizure frequency relative to the baseline.

**Fig. 3:**
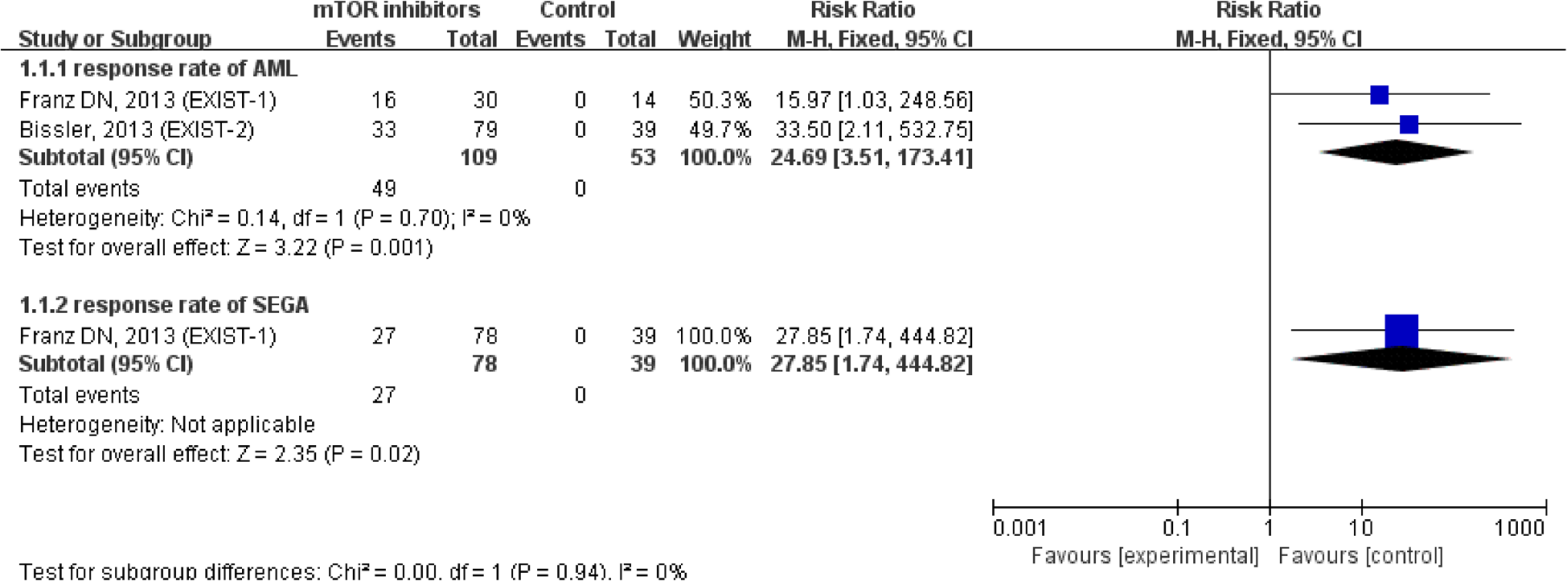
Forest plot of the response to tumor in TSC patients with or without mTOR inhibitors

Compared with placebo, mTOR inhibitors significantly reduced tumor volume in both AML (RR = 24.69, 95% CI = 3.51,173.41, *P* = 0.001) and SEGA (RR = 27.85, 95% CI = 1.74,444.82, *P* = 0.02). The pooled outcomes are shown in Fig. 3.

**Fig. 4:**
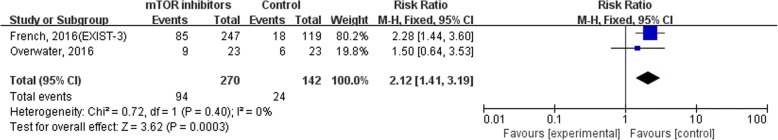
Forest plot of the response to seizure frequency in TSC patients with or without mTOR inhibitors

With regard to seizure frequency, the response rates were extracted from the two relevant studies [22]. Compared with placebo, mTOR inhibitors significantly reduced the seizure frequency (RR = 2.12, 95% CI = 1.41,3.19, *P* = 0.0003). The pooled outcomes are shown in Fig. 4.

#### Safety

**Fig. 5:**
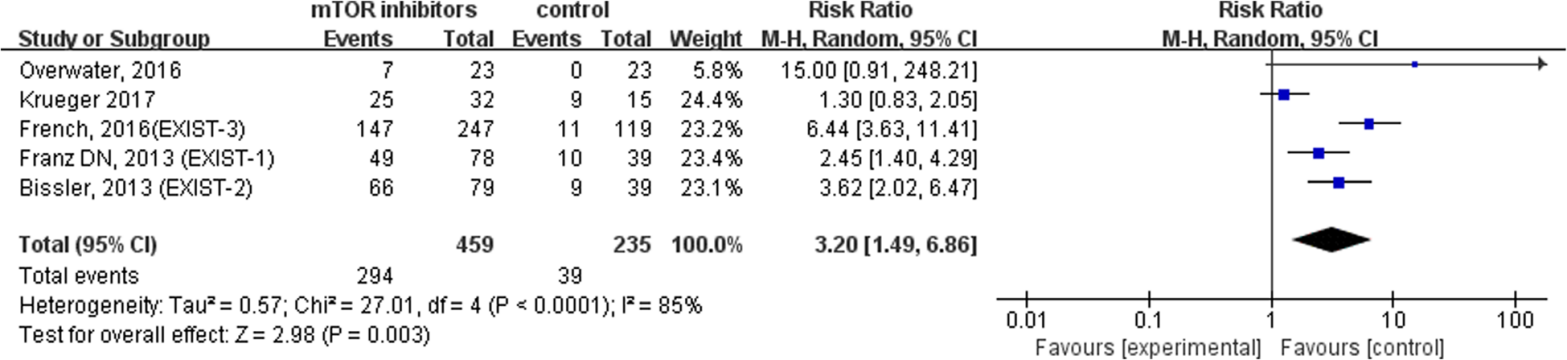
Forest plot of the incident of stomatitis in TSC patients with or without mTOR inhibitors

**Fig. 6:**
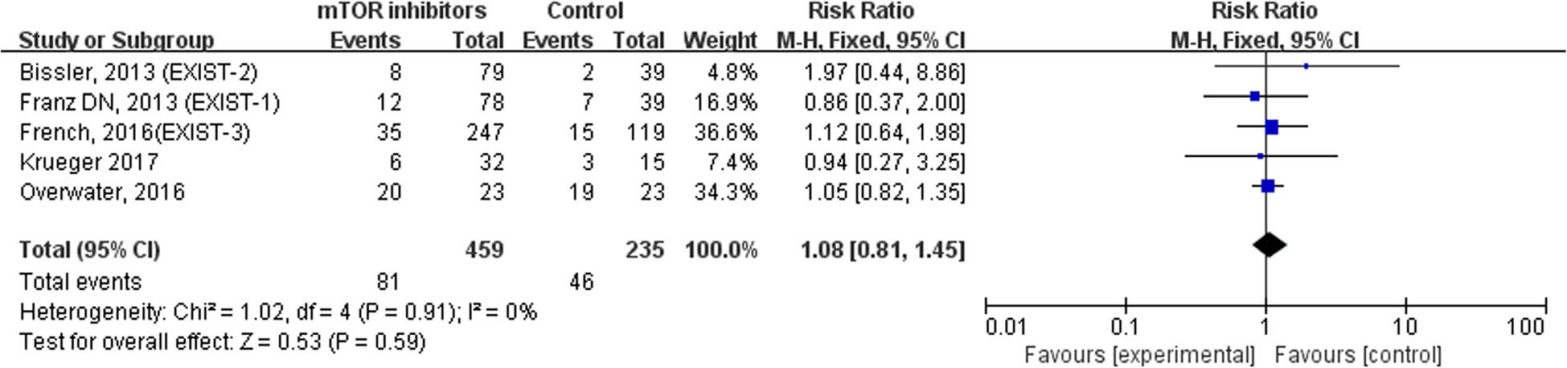
Forest plot of the incident of upper respiratory tract infection in TSC patients with or without mTOR inhibitors

**Fig. 7:**
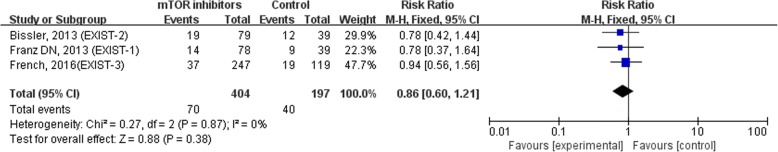
Forest plot of the incident of nasopharyngitis in TSC patients with or without mTOR inhibitors

According to the included studies and other reports, common adverse events of mTOR inhibitors associated with TSC therapy include stomatitis, upper respiratory tract infections, and nasopharyngitis except cough, vomiting and diarrhea. It has been noted that stomatitis includes all related terms, such as mouth ulceration, tongue ulceration, mucosal inflammation and gingival pain. We found that patients who received mTOR inhibitors had a higher risk of suffering stomatitis than those who did not (RR = 3.20, 95% CI = 1.49,6.86, *P* = 0.003). Heterogeneity of the effect measures regarding stomatitis was observed [*p* < 0.0001, I^2^ = 85%]. In contrast, the incidence of upper respiratory tract infections (RR = 1.08, 95% CI = 0.81,1.45, *P* = 0.59) and nasopharyngitis (RR = 0.86, 95% CI = 0.60,1.21, *P* = 0.38) were similar between the treatment group and the control group. All outcomes are shown in Figs. 5, 6 and 7.

## Discussion

This article was developed from the ever-increasing understanding of the relationship between TSC and mTOR inhibitors in scientific research. The results showed that the response rate in tumors (AML and SEGA) was significantly higher in the mTOR inhibitor-treated group than in the untreated group. Some recent open-label studies [27–29] have confirmed this finding. In addition, this study is the first to integrate clinical trial data to investigate the efficacy of mTOR inhibitors for the alleviation of seizures through a meta-analysis. Compared with participants with seizures who received the placebo, significantly more participants with seizures who received mTOR inhibitors experienced at least a 50% reduction in seizure frequency. An excluded study strongly supported this point with the result that all but 1 participant reported a ≥ 50% reduction in seizure frequency after 2 years [30]. However, due to limitations in research objects, experimental designs, outcome indications and original data from the clinical studies, we were unable to examine subgroups according to age and genetic characteristics.

Among the large number of AEs, we analyzed stomatitis, upper respiratory tract infections, and nasopharyngitis because they were the common AEs reported in the included studies. The above three AEs were more likely to be correlated with mTOR inhibitor therapy because they are not general as vomiting and diarrhea. Stomatitis, which occurred in approximately half the patients in the treatment group, including all the related terms, such as mouth ulceration, tongue ulceration, mucosal inflammation and gingival pain, was significantly associated with the use of mTOR inhibitors, unlike upper respiratory tract infections and nasopharyngitis. In our study, meta-analysis was also used for the first time to explore the correlation between the use of mTOR inhibitors and AEs. Approximately half of the patients in the treatment group had suffered stomatitis in our study population. However, most ADRs did not lead to level 3 or 4 toxicity. In a recent retrospective study, Krueger also reported that 40% of children developed stomatitis during mTOR inhibitor treatment [31]. According to the reviews by Martins [32] and Lo Muzio [33], grade 1 and grade 2 stomatitis occur more frequently in the first treatment cycle and seem to depend on the dose. Theoretically, mTOR inhibitors may induce an inflammatory reaction by inducing the release of keratinocyte cytokines, directly causing epithelial injury, which results in stomatitis [34]. However, the mechanism connecting mTOR inhibitors and stomatitis is more complex and requires further exploration and verification at the molecular and animal levels. In the real world clinical setting, patients should be instructed to maintain good oral hygiene and prevent stomatitis by the frequent non-alcoholic mouth wash or 0.9% salt water [35]. Local treatment with Sucralfate [36] or oral rinses with dexamethasone [35] will relieve the symptoms of stomatitis. Meanwhile, patients should avoid agents containing alcohol, hydrogen peroxide, iodine and thyme derivatives [35].

In addition to stomatitis, female amenorrhea events appeared both in the EXIST-1 and EXIST-2 trials, and the severity level was mostly grade 1 or 2. However, none of these patients reduced the dose they were receiving or discontinued treatment because of amenorrhea events, so there was no clear relationship between amenorrhea and drug therapy. However, more than 90% of women experienced amenorrhea in a two-year trial in China [29], which indicates that amenorrhea is considered as a potential risk and should be further investigated.

It should be noted that in the *Bissler* study [25], five patients (5 of 162) were diagnosed with sporadic lymphangioleiomyomatosis (not TSC), and they were analyzed for renal AML in this study. The *Franz* study evaluated seizure frequency in the form of the change from the baseline to week 24 as a key secondary endpoint. However, a large proportion of patients did not experience seizures at the baseline. Therefore, we did not include the results from the *Franz* study in the analysis of the seizure frequency [24]. In the *Overwater* study [22], the trial included sirolimus as an add-on treatment for epilepsy and did not include a placebo treatment, which may lead to bias. The Krueger study [26] used seizure frequency as one of the secondary outcomes, but the results were not reported in the paper or at *clinicaltrial.gov*. We also emailed the authors but received no response. Therefore, only the data regarding AEs were included and analyzed.

Most previous systematic reviews relating to rare diseases had small sample sizes, while our study included 671 patients in the meta-analysis, which was large enough to provide reliable evidence. To the best of our knowledge, our meta-analysis evaluating the efficacy and safety of mTOR inhibitors for TSC is more thorough than previous studies [20]. Prospective randomized controlled studies are generally regarded as the gold standard in the evaluation of therapeutic interventions; therefore, randomized controlled studies that met the eligibility criteria were included in this study. During the literature screening process, we excluded three RCTs for the following reasons. The *Koening* study [37, 38] performed in 2012 was excluded because it had a high risk of attrition bias, unclear allocation concealment, unclear random sequence generation, and used subjective improvement in skin lesions reported by patients. Although in *Randell* study [39], seizures were judged by a scale, which is a high-quality evaluation tool, no results were reported, and we received no response from the authors after emailing them. In addition, the report by *Xu Yan* [40] in 2016 lacked details regarding most aspects. Due to the bias in the evaluation of efficacy and safety caused by different administration methods, there were three RCTs that were not included in the analysis: one published results [41], one did not publish (NCT03140449), and one (NCT03363763) was in the recruitment phase. As a result, the RCTs included had high-quality, detailed data. However, the potential limitations of our review might include differences in the concomitant therapies used in the trials and the number of RCTs.

We also observed heterogeneity [I^2^ = 85%] in the meta-analysis of stomatitis. Limited to the inconsistency of original data in the included studies, a subgroup analysis could not be conducted. A sensitivity analysis was performed by removing each study in turn and recalculating the combined estimate for the remaining studies. The results revealed that the Krueger study is the main source of statistical heterogeneity in the meta-analysis of stomatitis. This may be due to design differences, including the inclusion criteria (limited enrollment to ≥6 years and IQ ≥ 60) and the follow-up duration (just 1 month) in that study. It should be noted that the heterogeneous results were not affected regardless of whether the random effect model or fixed effect model was used.

Additionally, there were several limitations of our meta-analysis. First, although the search strategy was comprehensive and the search results were updated compared with previous meta-analyses, there may still be some published and unpublished studies that were not included. Second, the treatment duration and the concomitant medications taken by patients were not consistent among the included trials, which might have led to bias. Third, stomatitis, which includes mouth ulceration, aphthous ulcer, tongue ulceration, mucosal inflammation, oropharyngeal pain and gingival pain, was not analyzed and discussed in detail according to the specific classification. Finally, it should be noted that a meta-analysis is a secondary study based on the literature and is inevitably affected by the quality of the literature. The number of trials included in this study was small when compared with systematic reviews of other common diseases. Based on the above limitations, it is necessary to take clinical situations into consideration when referring to the results of this meta-analysis.

Despite these limitations, the evidence clearly demonstrates the efficacy of mTOR inhibitors for the treatment of TSC. Further prospective studies with improved designs are needed to confirm these findings.

## Conclusions

In view of the efficacy and safety associated with reductions in tumor volume and seizure frequency in TSC patients, mTOR inhibitor is a good choice of medical therapy. The dose of mTOR inhibitors should be identified and incorporated into guidelines as soon as possible. Unlike upper respiratory tract infections and nasopharyngitis, the risk of grade 1 and 2 stomatitis seems to be increased by the administration of mTOR inhibitors. Further studies are needed to optimize the therapeutic strategy, including mTOR inhibitors, and to confirm the associated AEs.

## Abbreviations

AE: adverse event
AML: angiomyolipoma
FKBP12: FK 506-binding protein of 12 kDa
mTOR: mammalian target of rapamycin
mTORC1: mTOR complexes 1
RCTs: randomized controlled trials
RR: risk ratios
SEGA: subependymal giant cell astrocytoma
TSC: tuberous sclerosis complex
